# Multiplate Platelet Function Testing upon Emergency Room Admission Fails to Provide Useful Information in Major Trauma Patients Not on Platelet Inhibitors

**DOI:** 10.3390/jcm11092578

**Published:** 2022-05-05

**Authors:** Peter Pommer, Daniel Oberladstätter, Christoph J. Schlimp, Johannes Zipperle, Wolfgang Voelckel, Christopher Lockie, Marcin Osuchowski, Herbert Schöchl

**Affiliations:** 1 AUVA Trauma Centre Salzburg, Department of Anaesthesiology and Intensive Care Medicine, Academic Teaching Hospital of the Paracelsus Medical University, 5020 Salzburg, Austria; peter.pommer@stud.pmu.ac.at (P.P.); daniel.oberladstaetter@auva.at (D.O.); wolfgang.voelckel@auva.at (W.V.); christopher.lockie@auva.at (C.L.); 2 Paracelsus Medical University, 5020 Salzburg, Austria; 3 Ludwig Boltzmann Institute for Traumatology, The Research Centre in Cooperation with AUVA, 1020 Vienna, Austria; c.schlimp@gmx.at (C.J.S.); johannes.zipperle@trauma.lbg.ac.at (J.Z.); marcin.osuchowski@trauma.lbg.ac.at (M.O.)

**Keywords:** platelet dysfunction, trauma, polytrauma, TBI, Multiplate

## Abstract

Platelet dysfunction is a suggested driver of trauma-induced coagulopathy. However, there is still a paucity of data regarding the impact of injury pattern on platelet function and the association of platelet dysfunction on transfusion requirements and mortality. In this retrospective cohort study, patients were grouped into those with isolated severe traumatic brain injury (TBI group), those with major trauma without TBI (MT group), and a combination of both major trauma and traumatic brain injury (MT + TBI group). Platelet function was assessed by whole blood impedance aggregometry (Multiplate^®^, MP). Three different platelet activators were used: adenosine-diphosphate (ADP test), arachidonic acid (ASPI test), and thrombin activated peptide-6 (TRAP test). Blood transfusion requirements within 6 h and 24 h and the association of platelet dysfunction on mortality was investigated. A total of 328 predominantly male patients (75.3%) with a median age of 53 (37–68) years and a median ISS of 29 (22–38) were included. No significant difference between the TBI group, the MT group, and the MT + TBI group was detected for any of the investigated platelet function tests. Unadjusted and adjusted for platelet count, the investigated MP assays revealed no significant group differences upon ER admission and were not able to sufficiently predict massive transfusion, neither within the first 6 h nor for the first 24 h after hospital admission. No association between platelet dysfunction measured by MP upon ER admission and mortality was observed. Conclusion: Injury pattern did not specifically impact platelet function measurable by MP. Platelet dysfunction upon ER admission measurable by MP was not associated with transfusion requirements and mortality. The clinical relevance of platelet function testing by MP in trauma patients not on platelet inhibitors is questionable.

## 1. Introduction

Trauma-related haemostatic dysfunction, also termed trauma-induced coagulopathy (TIC), can be detected in approximately 25–35% of severely injured patients upon emergency room admission [1,2]. Early endogenous TIC is associated with an increased bleeding tendency, a higher rate of red blood cell (RBC) transfusion requirements, and an elevated incidence of secondary complications such as multi organ dysfunction syndrome or thromboembolism [3]. Despite enormous progress in the global understanding of TIC, the exact mechanisms behind this phenomenon are still not fully understood [4]. Multiple potential drivers of TIC including shock-related hypoperfusion, systemic fibrinolysis, endothelial cell and glycocalyx damage, an overwhelming inflammatory response, clotting factor activation, and platelet dysfunction have been identified [5].

Platelets play a fundamental role not only in primary haemostasis but also for innate immunity. Vascular injury and exposure of the subendothelial collagen is a strong platelet activator and the initial driver of primary clot formation. Thus, compromised platelet function contributes to poor clot quality and is associated with an exacerbated bleeding tendency and increased mortality [6].

In severely injured trauma patients, platelet function is profoundly disturbed by a variety of suggested mechanisms. However, the exact drivers behind trauma-induced platelet dysfunction are still under discussion. Following trauma, diminished response to several platelet agonists, proteolysis and internalisation of platelet surface receptors, inhibition of several calcium signalling pathways, and histone-related platelet ballooning have been reported [7]. Even after mild trauma, aggregometry testing revealed a poor response to multiple platelet agonists, and this finding was also associated with a poor outcome [8,9].

One important hinderance in studies focusing on platelet function is the lack of a “gold standard” point of care monitoring tool. All available devices such as TEG Platelet mapping^®^, Verifynow^®^, the Platelet Function Analyser PFA^®^100/200, or Multiplate^®^ (MP) were initially developed to examine platelet response to antiplatelet therapy, rather than a diagnostic instrument for the detection of bleeding risk.

Nevertheless, point-of-care platelet function testing in trauma patients has been recommended by the fifth update of the European guideline on management of major bleeding and coagulopathy following trauma [10].

There is ongoing debate if and to what extent different injury patterns affect the haemostatic function of platelets [11,12,13]. Numerous studies reported major platelet dysfunction following isolated traumatic brain injury (TBI) [12,14,15,16,17]. Moreover, an association between the severity of TBI and platelet dysfunction has been described [18]. In particular, a strong inhibition of the adenosine-diphosphate (ADP) and arachidonic acid pathway have been documented in TBI patients [19]. However, it is uncertain as to whether severe TBI affects platelet function to a higher extent compared to patients without brain tissue trauma.

In our level 1 trauma centre, the whole blood aggregometry (Multiplate^®^, Roche Diagnostics, France) was implemented in 2008 and is currently part of the standard coagulation panel for severely injured trauma patients. The MP analyser measures the aggregation of platelets in response to different agonists such as adenosine diphosphate (ADP-test), arachidonic acid (ASPI-test), collagen (COL-test), and thrombin-receptor-activating peptide 6 (TRAP-test) [20]. Thus, the device provides crude information about a potential inhibition of some relevant platelet activation pathways. A variety of animal and human studies revealed that platelet dysfunction, detected by MP, was associated with poor outcome [6,18,21].

The primary aim of the current study was to compare platelet function of patients with isolated traumatic brain injury (TBI), major trauma (MT) without TBI, and a combination of both MT + TBI, as measurable with MP. We hypothesised that isolated TBI does not impact platelet function measurable with MP to a higher extent than in patients without brain tissue damage. Furthermore, we investigated the predictive value of three different MP tests, run upon an emergency room admission, for massive transfusion requirement and mortality.

## 2. Materials and Methods

### 2.1. Study Design

**Figure 1:**
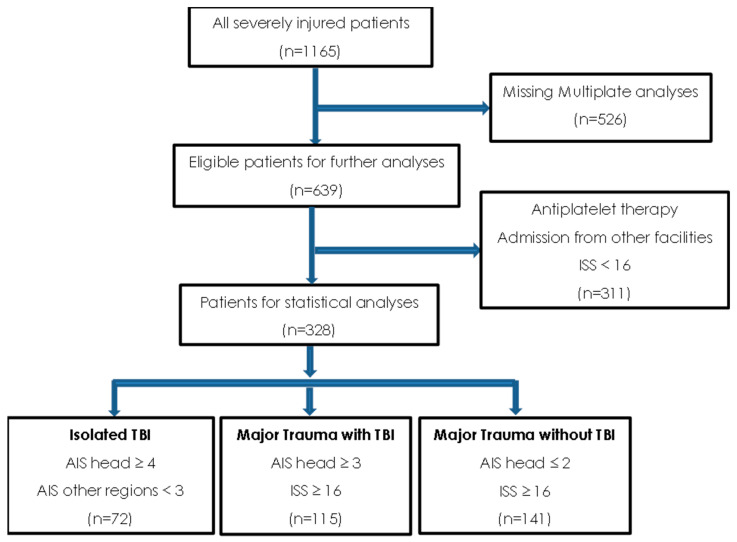
Included and excluded patients with multiplate test results upon emergency room admission.

Following the approval of the local ethics committee of the government of Salzburg (EK number: 1143/2021), this retrospective data analysis of prospectively gathered data from severely injured trauma patients admitted to the AUVA Trauma Centre Salzburg, a certified level 1 trauma hospital, was conducted. Data were gathered between June 2012 and July 2021 and were retrospectively analysed. Since 2012, data from severely injured trauma patients have been collected prospectively and transferred to the “German Trauma Registry of the DGU”. This is one of the largest trauma databases worldwide, which incorporates almost 450,000 patients. For the current study, all trauma patients ≥16 years who were directly admitted from the site of the accident to the emergency room in which MP platelet testing was performed were eligible for analyses. Exclusion criteria were patients with an age < 16 years, patients transported from other facilities, pregnant patients, patients with minor injuries (ISS < 9), and those with known antiplatelet therapy and missing platelet function tests. (Figure 1).

Demographic and clinical and laboratory date were extracted from (i) the ICU COPRA 6^®^ database, (ii) and hospital length of stay (LOS) was retrieved from the hospital information database ASTRA^®^. (iii) Allogeneic blood transfusions were collected from a dedicated database (Datalab^®^, Graz, Austria) where all transfusions have to be documented.

### 2.2. Laboratory Measurements, Standard Coagulation Tests, and Multiplate Analyses

The routine laboratory analyses panel for severely injured trauma patients consists of full blood cell count, blood gas analyses, lactate, and the pro-inflammatory cytokine interleukin (IL)-6.

Standard coagulation tests such as prothrombin time index (PTI), international normalised ratio (INR), activated partial thromboplastin time (aPTT), and fibrinogen concentration were analysed in the central laboratory. Visco-elastic testing (ROTEM^®^ or ClotPro^®^) was performed as point of care measurements in the emergency room.

### 2.3. Multiplate Analyses

Whole blood aggregometry platelet function tests were run on a Multiplate^®^ device (Roche^®^, Grenzach-Wyhlen, Germany). Multiplate is a 5-channel platelet function analyser which measures platelet function in response to the addition of different platelet activators [22]. In the majority of the patients, a 20 G arterial line was placed, and a blood sample of 3 mL was collected in hirudin-containing tubes (S-Monovette Hirudin, Sarstedt^®^, Nürmbrecht, Germany). After a short resting time, 0.3 mL blood was pipetted in a cup and diluted with another 0.3 mL of normal saline into a special test cell. In the blood sample, two silver-coated copper wires were immerged, and a constant current was applied between the wires. Following the addition of platelet activators, platelets adhered to the surface of the wire, which raised the resistance of the applied current. An increase in the impedance is displayed as a curve over time. The AUC (area under the curve = aggregation units against time in minutes) represents the function of the platelets [22].

In order to investigate the impact of trauma on platelet function, three different platelet agonists were applied: (i) adenosine-diphosphate test (ADP-test, manufacturers reference range 53–122 U), arachidonic acid test (ASPI-test, manufacturers reference range 75–136 U), and thrombin receptor activating peptide 6-test (TRAP-test, manufacturers reference range 94–156 U). All coagulation analyses were performed within the first hour after ER admission.

As platelet count might have an impact on the results, the AUC was adjusted for platelet count. AUC was divided by platelet number and multiplied by 100 (adjusted AUC: AUC/platelet count × 100).

In order to compare platelet dysfunction of isolated TBI, MT patients without TBI, and a mixed population of major trauma and TBI (MT + TBI), 3 different groups of patients were established: (i) patients with isolated TBI, defined as an abbreviated injury score (AIS) for the head > 3 and an AIS of other body regions of <3; (ii) major trauma patients without TBI (AIS head < 3) and an AIS of other body regions of >3; and (iii) major trauma patients with TBI (AIS head > 3 and a total ISS > 15). Massive transfusion was classified as a transfusion of ≥10 packed red blood cells within the first 24 h [23].

### 2.4. Statistical Analysis

All data are presented as median and interquartile ranges (25% and 75%). The distribution of data was assessed by applying the Shapiro–Wilk test. For non-parametric data, the Mann–Whitney test and Kruskal–Wallis tests were used. For normally distributed data, Student’s *t*-test and one-way ANOVA tests were performed.

Correlation coefficients for ADP test, ASPI test, and TRAP test vs. RBC transfusion within 6 h and 24 h were calculated using the Spearman nonparametric test and linear regression models. Correlations were classified as weak (coefficient values 0.20–0.39), moderate (0.40–0.59), strong (0.60–0.79), or very strong (≥0.80).

The values of different multiplate tests regarding the prediction of massive transfusion receiver operating characteristic curves (ROC) were calculated.

A *p*-value < 0.05 was defined as statistically significant. Statistical calculations were performed using GraphPad Prism (Version 9.3.1., GraphPad Software, La Jolla, CA, USA).

## 3. Results

### 3.1. Demographic and Clinical Data

Between June 2012 and July 2021, a total of 328 predominantly male (75.3%) subjects fulfilled the inclusion criteria (Figure 1). The mean age was 53 (37–68 IQR) years, the median ISS was 29 (22–38). According to the predefined criteria, there were 72 patients in the isolated traumatic brain injury group (TBI group), 141 patients in the group of major trauma without TBI (MT group), and 115 patients in the group of major trauma with additional TBI (MT + TBI group). The demographic and clinical data upon ER room admission are outlined in Table 1.

### 3.2. Laboratory Data

Laboratory data upon ER room admission are depicted in Table 2. Significant group differences were observed for all shown parameters.

### 3.3. Whole Blood Aggregometry

None of the whole blood platelet aggregometry tests demonstrated a significant difference among groups. None of the applied tests discriminated isolated TBI from MT and/or MT + TBI patients. Similar results were observed after adjusting for the platelet count Table 3.

Moreover, we could not demonstrate any significant impact of a shock-related hypoperfusion, assessed by base excess on platelet function measured by MP. Patients with a base excess ≥−6 mmol/L and <−6 mmol/L revealed similar AUCs unadjusted and adjusted for platelet count (data not presented).

**Figure 2:**
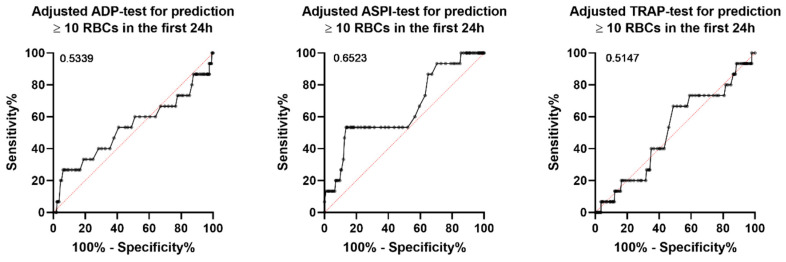
ROC curves for transfusion of ≥10 RBCs within 24 h adjusted for platelet count. ADP, adenosine diphosphate; ASPI, arachidonic acid test; TRAP, thrombin-receptor-activating peptide; ROC, receiver operating characteristics.

ROC analyses for the prediction of massive transfusion within 6 h or 24 h also demonstrated low area under the curves (AUC) for all investigated MP tests. An adjustment for the platelet count had no further impact on the results (Figure 2).

**Figure 3:**
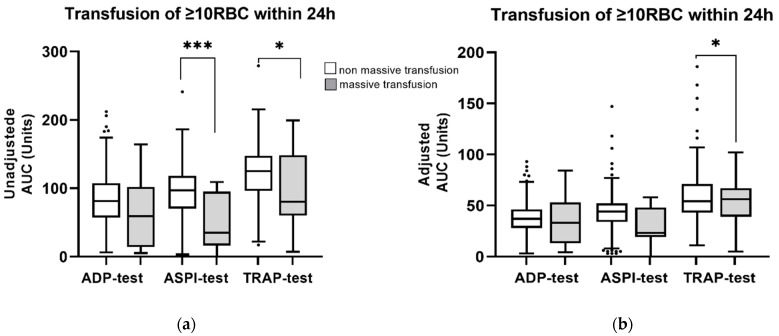
(**a**,**b**) Multiplate tests and analyses for transfusion of ≥10RBCs within 24 h after hospital admission, unadjusted and adjusted for platelet count. White square, non-massive transfusion; grey square, massive transfusion; ADP, adenosine diphosphate; ASPI, arachidonic acid test; TRAP, thrombin-receptor-activating peptide. AUC, area under curve; RBC, red blood cell; * *p* < 0.05; *** *p* < 0.001; no indication = not significant. Data are presented as box and whisker plots (Tukey). Student’s *t*-test or Mann–Whitney rank sum test was used as appropriate for between-group comparisons.

When comparing massive transfusion within 24 h, significant differences of the AUC were detected for the unadjusted ASPI test and the TRAP test. After adjusting for the platelet count, only the TRAP test remained significant. However, the differences between massive and non-massive transfusion were small (Figure 3).

**Figure 4:**
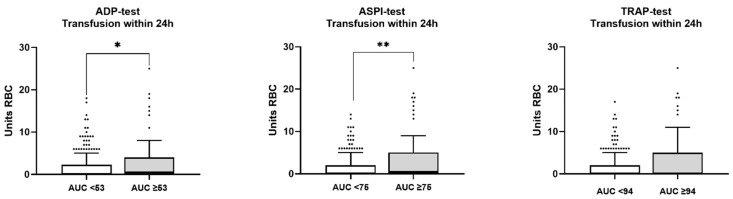
Multiplate tests below and within reference range and RBC transfusion in the first 24 h after hospital admission. White, normal reference range; grey, lower reference range. ADP, adenosine diphosphate; ASPI, arachidonic acid test; TRAP, thrombin receptor activating peptide; AUC, area under curve; RBC, red blood cells. * *p* < 0.05; ** *p* < 0.01; no indication = not significant. Data are presented as box and whisker plots (Tukey). Student’s *t*-test or Mann–Whitney rank sum test was used as appropriate for between-group comparisons.

No correlations between any of the applied MP tests and RBC transfusion requirement could be detected (data not shown). Patients with MP test results below reference ranges compared with normal limits revealed higher transfusion requirements in the ADP test for the first 24 h and in the ASPI test for 6 h and 24 h (*p* < 0.05 and <0.01, respectively). No differences were observed for the TRAP test (Figure 4).

Furthermore, no significant differences in platelet dysfunction measurable by MP among groups regarding survival were found. Both the ADP test and the ASPI test revealed a significant difference only for the MT group. After adjusting for the platelet count, no significant difference in any of the groups was found (Figure S1).

ROC analyses for mortality demonstrated low AUCs and ranged between 0.512 and 0.618 (Figure S2).

## 4. Discussion

In this cohort of well-matched trauma patients, no significant difference among the group of isolated TBI patients, MT without TBI, and the MT + TBI combination was detected for any of the investigated variables. Unadjusted and adjusted for the platelet count, the MP assays ADP test, ASPI test, and TRAP tests revealed no significant differences among groups upon ER admission. Moreover, the investigated MP tests failed to sufficiently predict massive transfusion, neither within the first 6 *h* nor for the first 24 h after hospital admission. No association between platelet dysfunction and mortality was observed.

Platelet function testing is a well-established method for assessing the effect of platelet inhibitors such as aspirin or ADP receptor blocking substance on the platelet function [24]. The gold standard for platelet function testing is the light transmission aggregometry [25]. This is a labour-intensive method that requires a profound expertise and therefore it is only available in specialised centres. Multiplate is increasingly used in patients on P2Y12 receptor inhibitors after percutaneous coronary intervention [26]. Moreover, preoperative platelet function analyses have been established in some centres and are routinely utilised for the before–after cardiac surgery measurements [27]. Despite several positive results, the overall usefulness of these devices for detection of trauma-induced platelet inhibition remains uncertain.

### 4.1. Platelet Function and Injury Pattern

A low platelet count in severely injured patients is associated with poor outcome in both isolated TBI and polytrauma [28,29]. However, the platelet count does not provide any meaningful information about the platelet function. Numerous animal and human studies reported significant platelet function impairment following isolated TBI [15,16,18,19]. Moreover, an association between the severity of TBI and platelet dysfunction has been described. A mild TBI compromised platelet function to a significantly lower extent compared to severe TBI [18,19,30]. Guillotte et al. assessed platelet dysfunction by TEG PM (TEG^®^6S device with Platelet mapping^®^ cartridge) and observed that severe and moderately isolated TBI were associated with a higher ADP inhibition compared to mild TBI. Moreover, severe TBI resulted in a significantly higher ADP inhibition compared to polytrauma patients. Arachidonic acid inhibition was comparable between both groups [18]. This finding contrasts the results of the current study; all three investigated MP assays failed to show any significant inter-group differences. Our data revealed that major trauma with associated TBI does not impair platelet function measurable by MP more than other injury patterns. Moreover, MP platelet function testing upon admission did not correlate with the injury severity.

Hypovolemic shock has been identified as an important driver of TIC [5]. However, the impact of hypoperfusion on platelet dysfunction has to be elucidated. In a prospective study on 101 trauma patients, Kutcher et al. reported an association between base deficit and impaired platelet aggregation [6]. No relationship between the severity of different shock states as reflected by base excess and lactate, and diminished platelet aggregation could be confirmed by the current study. In alliance with our findings, Windeløv et al. also reported no association between impaired platelet function and base deficit [31].

### 4.2. Platelet Function and Transfusion Requirements

Platelet dysfunction is a suggested driver of TIC. However, it is uncertain if and to what extent diminished platelet function contributes to bleeding tendency and transfusion requirements. For trauma patients and patients with isolated TBI, there is still a paucity of evidence that any platelet function testing provides useful information regarding a potential bleeding risk. Stettler et al. reported prospectively collected data from 303 trauma patients in whom TEG PM was performed upon ER admission. The percentage of ADP receptor inhibition was neither predictive for massive transfusion nor for platelet transfusion. This finding is in alliance with the results of the current study. No association between the intensity of MP platelet dysfunction and RBC transfusion requirements could be detected [32].

### 4.3. Platelet Function and Mortality

Several studies reported an association between the extent of platelet dysfunction and a poor outcome following trauma. However, the results of these studies were not conclusive. Solomon et al. demonstrated that in major trauma patients, upon emergency room admission, MP ADP test and TRAP test were significantly lower in non-survivors compared to survivors [21]. Kutcher et al. reported that 46% of the investigated subjects had decreased platelet response to at least one platelet agonist upon emergency room admission, and 91% experienced platelet function impairment during their ICU stay. On admission, platelet dysfunction was associated with an almost 10-fold higher early mortality compared to normal platelet response [6]. In TBI patients, TEG PM showed significant correlations between the degree of ADP receptor inhibition and risk of mortality [19]. In contrast, Guillotte and co-workers found no association between the intensity of TEG PM ADP inhibition and in-hospital mortality [18]. This corresponds well with the findings of our study. Unadjusted for the platelet count, we found a statistically significant difference in ADP test and ASPI test between survivors and non-survivors. However, after adjusting the AUC for the platelet count, such differences were no longer observed.

## 5. Limitations

Several limitations of this study have to be considered. This is a retrospective analysis of prospectively collected data of trauma patients upon ER admission. It cannot be ruled out that some of the included patients were on platelet inhibitors such as aspirin and/or non-steroidal anti-inflammatory drugs prior to injury, which might have impacted platelet function. In the current study, we investigated our patients with the multiplate analyser using three different platelet activators. The results could be substantially different if other platelet function testing (e.g., light transmission aggregometry, TEG PM, PFA 100/200) would have been used. It cannot be excluded that platelet function tests investigating alternative pathways could potentially reveal different findings. Most importantly, previous studies revealed only a poor correlation between these different platelet function tests in trauma patients [33].

## 6. Conclusions

Although point-of-care platelet function testing in trauma patients has been recommended by the European guideline on management of major bleeding and coagulopathy following trauma, the current study demonstrated that injury pattern and shock severity did not impact platelet dysfunction assessed by three different multiplate tests. Moreover, no association between platelet dysfunction and transfusion requirements and mortality was apparent. Thus, the platelet function testing by MP upon ER admission in patients not on platelet inhibitors is not informative for subsequent clinical decision making in our trauma centre. The additional considerable resources spent on costs for the platelet function testing are not justified in trauma patients nor on platelet inhibitors.

## Figures and Tables

**Table 1:** Demographics, clinical data, and injury scores upon emergency room admission.

	TBI Group	MT Group	MT + TBI Group	*p*-Value
Number of patients	72	141	115	
Age, (years)	58 (34–73)	52 (38–66)	52 (36–67)	ns
Male, *n* (%)	51 (70.8%)	101 (71.6%)	95 (82.6%)	ns
Syst. BP, (mmHg)	135.5 (110–160) ^a,b^	120 (96–149)	118 (98–142)	0.0012
HR, (bpm)	82.5 (68–102) ^a,b^	94.5 (80–113)	95 (80–116)	0.0048
SpO_2_ (%)	98 (93–99)	96.5 (91–99)	97 (93–99)	ns
ISS	25 (24–28)	25 (20–34)	34 (27–45) ^a,c^	<0.0001
NISS	34 (25–45)	33 (24–43)	43 (34–59) ^a,c^	<0.0001
GCS	8 (4–18)	15 (13–15) ^b,c^	6 (3–12)	<0.0001
AIS—head	5 (4–5) ^a,b;^	0 (0–1)	4 (3–5) ^c^	<0.0001
AIS—thorax	0 (0–0) ^a,b^	3 (2–4)	3 (2–4)	<0.0001
AIS—abdomen	0 (0–0) ^a,b^	2 (0–3)	0 (0–2) ^c^	<0.0001
AIS—extremities	0 (0–0) ^a,b^	3 (2–4)	2 (0–3)	<0.0001
≥6 RBC within 6 h, *n* (%)	1 (1.4) ^a,b^	19 (13.5)	10 (8.7)	0.0148
≥10 RBC within 24 h, *n* (%)	1 (1.4) ^a,b^	13 (9.2)	3 (2.6)	0.0155
ICU length of stay (days)	10 (5–16) ^a,b^	9 (5–20)	15 (7–24)	0.0331
Hospital length of stay (days)	14 (7–24) ^a,b^	22 (12–37)	24 (10–39)	0.0006
Mortality, *n* (%)	19, (26.0%)	14, (9.9)	20, (17.4)	0.0077

TBI, traumatic brain injury; MT, major trauma; Syst. BP, systolic blood pressure; HR, heart rate per minute; SpO_2_, peripheral oxygen saturation; ISS, injury severity score; NISS, new injury severity score; GCS, Glasgow coma scale; AIS, abbreviated injury scale; RBC, red blood cells; ICU, intensive care unit; ^a^ TBI vs. TBI + MT; ^b^ TBI vs. MT; ^c^ MT vs. TBI + MT, all *p* < 0.05.

**Table 2:** Laboratory data measured upon ER admission.

	TBI Group	MT Group	MT + TBI Group	*p*-Value
Hemoglobin (g/dL)	13.3 (11.8–14.2) ^b^	12.4 (10.6–14)	12.5 (11.3–13.9)	0.0271
Platelet count (G/L)	213 (167–244) ^b^	233 (193–281)	210 (178–257)	0.0148
Lactate (mmol/L)	1.7 (1.2–2.8) ^b^	2.5 (1.6–3.6)	2.1 (1.4–3.1)	0.0045
BE (mmol/L)	−1.95 (−3.9–−0.9) ^a,b^	−2.7 (−4.9–−1.4)	−3.2 (−4.8–−1.3)	0.0281
pH	7.37 (7.32–7.41) ^a,b^	7.34 (7.28–7.38)	7.34 (7.3–7.38)	0.0098
IL-6 (ng/mL)	38 (14–67) ^a,b^	138.9 (69–271)	144 (49–273)	<0.0001
Standard coagulation tests				
PT (%)	92 (79–103) ^a,b^	84 (70–95)	81 (64–92)	0.003
aPTT (s)	28 (26–30)	27 (25–30) ^c^	29 (26–33)	0.0076
Fibrinogen (mg/dL)	267 (227–313) ^a,c^	255 (209–315)	233 (187–277)	0.0007

ER, emergency room, IL-6, interleukin 6; PT, prothrombin time; aPTT, activated partial thromboplastin time; TBI, traumatic brain injury; MT, major trauma; BE, base excess; Kruskal–Wallis test and Dunn’s multiple comparison, ^a^ TBI vs. TBI + MT; ^b^ TBI vs. MT; ^c^ MT vs. TBI + MT, all *p* < 0.05.

**Table 3:** Multiplate test results upon ER admission adjusted and unadjusted for platelet count.

Multiplate Tests	TBI Group	MT Group	MT + TBI Group	*p*-Value
Number of patients with test results below reference ranges				
ADP test, *n* (%)	12 (16.7)	26 (18.4)	26 (22.6)	0.5553
ASPI test, *n* (%)	21 (29.2)	33 (23.4)	36 (31.3)	0.3458
TRAP test, *n* (%)	17 (23.6)	32 (26.7)	29 (25.2)	0.8941
Unadjusted for platelet count (AUC)				
ADP test	83 (55–106)	81 (58–107)	77 (55–107)	0.8890
ASPI test	93 (61–121)	99 (74–120)	96 (61–117)	0.4694
TRAP test	126 (95–148)	126 (97–148)	121 (90–145)	0.9020
Adjusted for platelet count (AUC/platelet count × 100)				
ADP test	39 (27–51)	34 (26–45)	38 (28–46)	0.1882
ASPI test	43 (33–56)	43 (33–50)	45 (33–54)	0.3941
TRAP test	57 (45–78) ^b^	52 (40–68)	55 (41–72)	0.0430

ER, emergency room; TBI, traumatic brain injury; MT, major trauma; ADP, adenosine diphosphate; ASPI, arachidonic acid test; TRAP, thrombin-receptor-activating peptide; AUC, area under the curve; Kruskal–Wallis test and Dunn’s multiple comparison, ^b^ TBI vs. MT *p* < 0.05.

## Data Availability

The data presented in this study are available on request from the authors.

## Supplementary Materials

The following supporting information can be downloaded at: https://www.mdpi.com/article/10.3390/jcm11092578/s1, Figure S1: Survivors vs. non-survivors according to different injury pattern, Figure S2: ROC for mortality unadjusted and adjusted for platelet count.
