# Supplementary material for: Multiplate Platelet Function Testing upon Emergency Room Admission Fails to Provide Useful Information in Major Trauma Patients Not on Platelet Inhibitors
Source: J Clin Med. 2022 May 5;11(9):2578. doi: 10.3390/jcm11092578 (PMC9100631; doi:10.3390/jcm11092578)
Supplement: Supplementary file 1 [file jcm-11-02578-s001.zip › jcm-1690700-supplementary.pdf]

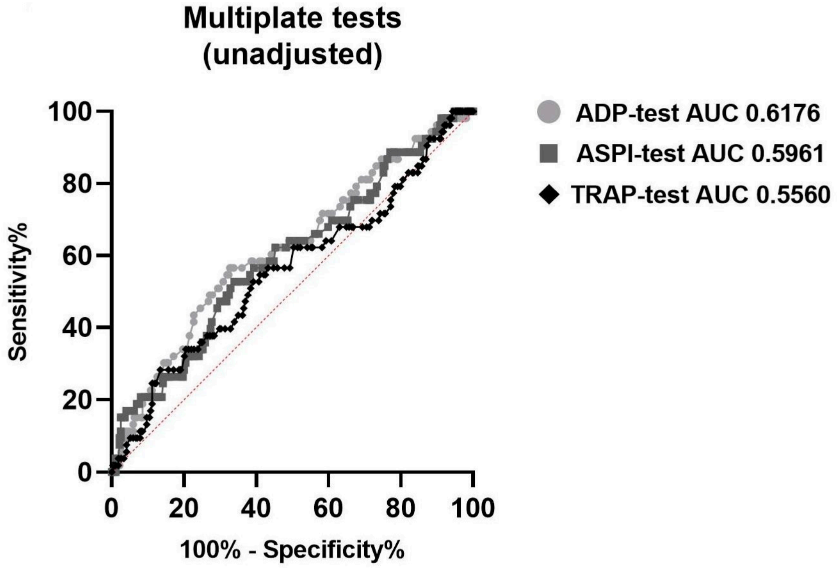

Figure S1. Survivors vs. non-survivors according to different injury pattern.

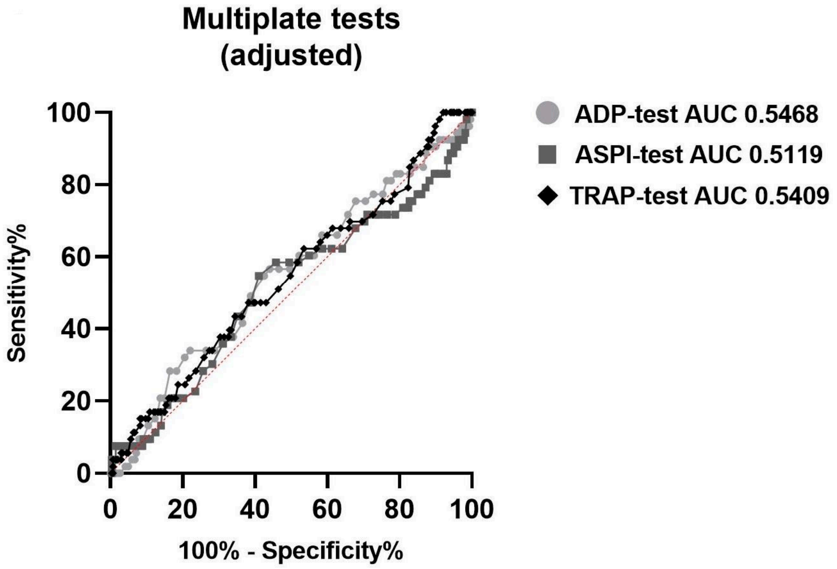

Figure S2. ROC for mortality unadjusted and adjusted for platelet count.
